# Atrial arrhythmias following lung transplant: a single pediatric center experience

**DOI:** 10.3389/fped.2023.1161129

**Published:** 2023-06-23

**Authors:** Jordan Sill, Shankar Baskar, Huaiyu Zang, David Spar, Ilias Iliopoulos, David L. S. Morales, Don Hayes, Wonshill Koh

**Affiliations:** ^1^ Heart Institute, Cincinnati Children’s Hospital Medical Center, Cincinnati, OH, United States; ^2^ Department of Pediatrics, University of Cincinnati College of Medicine, Cincinnati, OH, United States; ^3^ Division of Pediatric Cardiothoracic Surgery, Cincinnati Children’s Hospital Medical Center, University of Cincinnati College of Medicine, Cincinnati, OH, United States; ^4^ Division of Pulmonary Medicine, Cincinnati Children’s Hospital Medical Center, Cincinnati, OH, United States

**Keywords:** atrial arrhythmia, lung transplantation, post-operative complication, pediatric center, outcome

## Abstract

**Background:**

Outcomes after lung transplant (LTx) in children have slowly improved. Although atrial arrhythmia (AA) is a common and adverse complication following LTx among adults, there is limited data on pediatric recipients. We detail our pediatric single-center experience while providing further insights on occurrence and management of AA following LTx.

**Methods:**

A retrospective analysis of LTx recipients at a pediatric LTx program from 2014 to 2022 was performed. We investigated timing of occurrence and management of AA following LTx, and its effect on post-LTx outcome.

**Results:**

Three out of nineteen (15%) pediatric LTx recipients developed AA. The timing of occurrence was 9–10 days following LTx. Those patients in the older age group (age >12 years old) were the only ones who developed AA. Developing AA did not have a negative effect on hospital stay duration or short-term mortality. All LTx recipients with AA were discharged home on therapy that was discontinued at 6 months for those who was on mono-therapy without recurrence of AA.

**Conclusions:**

AA is an early post-operative complication in older children and younger adults undergoing LTx at a pediatric center. Early recognition and aggressive management can mitigate any morbidity or mortality. Future investigations should explore factors that place this population at risk for AA in order to prevent this complication post-operatively.

## Introduction

Since the first successful pediatric lung transplant (LTx) in 1987, the number of pediatric LTxs has increased with more than 2,000 LTxs performed globally (1–6). Although the overall survival after pediatric LTx has markedly improved in the past 10 years, the survival rate remains inferior to those of adult LTx recipients and pediatric heart transplant recipients (4–6). Key risk factors that influence early post-LTx outcomes include donor and recipient matching, intraoperative course, and postoperative complications including primary graft dysfunction and allograft failure (1–6). The early post-operative course for a child after LTx requires specialized teams with vast experience and familiarity in managing these complex pediatric patients (7). One of commonly recognized postoperative complications in adults after LTx is atrial arrhythmias with reported prevalence up to 50%, leading to increased length of hospital stay and mortality (8–13). Compared to adults, published data on atrial arrhythmias among children and young adults following LTx are sparse with reported prevalence rates of 11%–20% across pediatric LTx centers (14–16). Given the changing pediatric LTx landscape, optimization of clinical management of pediatric LTx recipients is a priority, especially in the post-operative time period until discharge (17, 18). Long-term prognosis for children who survive the early LTx course is favorable and comparable to or exceeds adult outcomes following LTx (19, 20).

We hypothesized that the occurrence of atrial arrhythmias among pediatric populations is less than among adult population, but we recently experienced more atrial arrhythmias at our institution following LTx. With data and studies of LTx in pediatric centers being far less numerous than those in adult centers, we believe that our data and single-center experience would increase awareness and better define the impact of atrial arrhythmias in these children as well as young adults and initiate discussions on best approaches to management.

## Materials and methods

### Study design and patient selection

The study was approved by the Institutional Review Board at Cincinnati Children's Hospital Medical Center (CCHMC). All patients who underwent LTx from January 2014 to December 2022 at CCHMC were identified. For statistical analysis, only patients under the age of 18 were included. Two patients with a combined heart and lung transplant were excluded. All data was extracted from the hospital electronic medical records including patient demographics, operative report, echocardiogram, and electrocardiography. All patients were monitored in the cardiac intensive care unit (CICU) and cardiology step-down unit with continuous telemetry in the post-operative period.

### Statistical analysis

Medians with 25–75th percentiles, or frequencies with percent of total, were used to describe patient demographics and clinical characteristics. The comparisons between patients with and without atrial arrhythmias were tested by Wilcoxon rank-sum test for continuous variables and Fisher's exact test for categorical variables. *P*-values < 0.05 were considered statistically significant. All statistical analyses were performed using the R statistical program (version 3.6.1, https://www.r-project.org/).

## Results

### Patient characteristics

A total of nineteen patients under the age of 18 underwent LTx (all bilateral) at CCHMC during the study period (Table 1). The median age of the study cohort was 9 years of age. There were 10 males (52%). The median CICU and total hospital stays were 8 and 9 days, respectively for those with and without atrial arrhythmia. Six patients were admitted prior to LTx with all six requiring mechanical ventilation prior to LTx. There were three patients bridged to LTx on veno-venous (VV) extracorporeal membrane oxygenation (ECMO). The most common underlying etiologies for LTx were cystic fibrosis (CF) (26%) and primary pulmonary hypertension (PPH) (26%). There were one (5.3%) in-hospital mortality following LTx and three (16%) 1-year post-LTx mortalities.

**Table 1:** Patient characteristics for with and without atrial arrhythmias (AA).

Variable	Overall *N* = 19	Group		*p*-value
		Atrial tachycardia *N* = 3	No atrial tachycardia *N* = 16	
Age at Ltx (years)	9.00 (1.83, 14.50)	15.00 (14.00, 16.50)	5.50 (1.56, 14.00)	0.07
Mechanical ventilation following Ltx (days)	3.00 (1.00, 12.00)	1.00 (1.00, 2.50)	3.50 (1.00, 14.25)	0.3
ICU stay (days)	8.00 (6.00, 21.00)	8.00 (7.50, 19.00)	9.00 (5.75, 18.00)	0.7
Total hospital stay (days)	19.00 (15.00, 125.50)	97.00 (58.50, 155.00)	17.50 (14.75, 97.75)	0.2
Inotrope support (days)	2.00 (2.00, 4.50)	2.00 (1.50, 3.50)	2.00 (2.00, 4.25)	0.6
Bypass time (mins)	366.00 (314.00, 403.00)	405.00 (385.00, 475.00)	360.00 (300.25, 401.00)	0.2
Ischemic time (mins)	393.00 (376.25, 426.75)	401.00 (300.50, 415.50)	391.00 (377.50, 424.50)	>0.9
Intubated prior	6 (32%)	1 (33%)	5 (31%)	>0.9
Admitted prior to TXP	6 (32%)	1 (33%)	5 (31%)	>0.9
ECMO bridge to LTx	3 (16%)	1 (33%)	2 (12%)	0.4
Hypertension	2 (11%)	0 (0%)	2 (12%)	>0.9
Diagnosis				0.9
Alveolar capillary dysplasia	2 (11%)	0 (0%)	2 (12%)	
CF	5 (26%)	1 (33%)	4 (25%)	
PPH	5 (26%)	0 (0%)	5 (31%)	
FLNA mutation	2 (11%)	0 (0%)	2 (12%)	
ILD	1 (5.3%)	1 (33%)	0 (0%)	
Cystic bullous lung disease	1 (5.3%)	1 (33%)	0 (0%)	
Other	3 (16%)	0 (0%)	3 (19%)	
In-hospital mortality	1 (5.3%)	0 (0%)	1 (6.2%)	>0.9
1-yr post LTx mortality	3 (16%)	0 (0%)	3 (19%)	>0.9

Categorical variables as *n* (%) and continuous variable as median (25–75th percentiles).

### Atrial arrhythmias following lung transplant

We identified three (15%) patients under the age of 18 who developed atrial arrhythmias following LTx (Table 1). The median age for those with atrial arrhythmia was 15 years compared to 5.5 years for those without atrial arrhythmia for all patients (*p*-value 0.07). All three patients who developed atrial arrhythmia had normal biventricular function with trivial tricuspid regurgitation on post-LTx echocardiogram with no subsequent change in ventricular function even with atrial arrhythmia. The patients without atrial arrhythmia had mildly decreased to normal right ventricular function, normal left ventricular function, and trivial to mild tricuspid regurgitation on echocardiograms following LTx. All patients had evidence of right ventricular hypertension based on a septal geometry on echocardiogram prior to LTx. No patient had a history of atrial tachycardia prior to LTx. Incidence of atrial arrhythmia did not have a statistically significant effect on the number of days of mechanical ventilation following LTx, duration of CICU stay following LTx, or total hospital stay. In addition, atrial arrhythmia had no statistically significant impact on in-hospital or 1-year post-LTx mortality.

**Figure 1:**
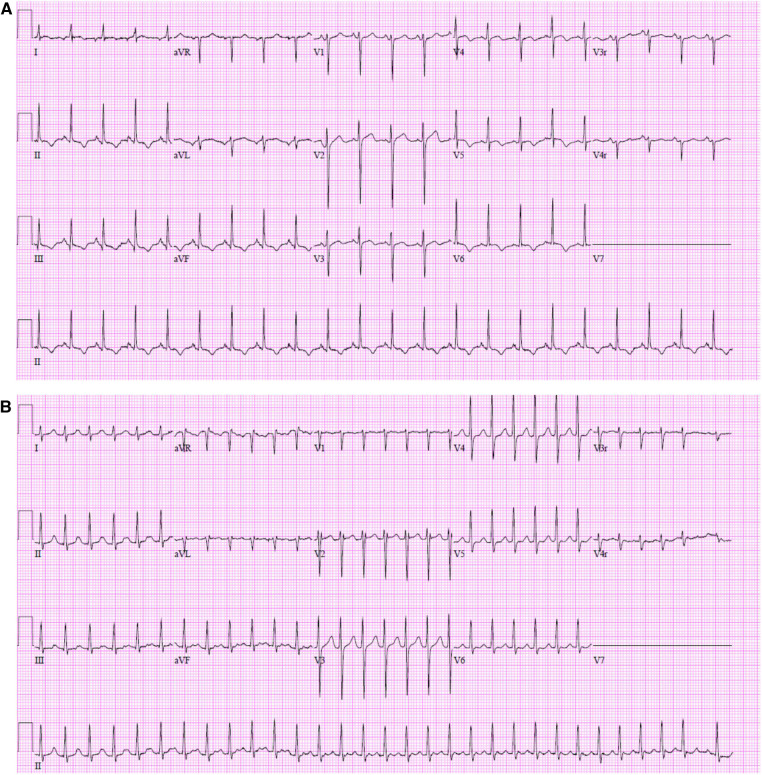
(**A**) Focal automatic atrial tachycardia. (**B**) Focal re-entrant atrial tachycardia.

In our cohort of patients, the post-operative period for developing atrial arrhythmias was 9–10 days following LTx and all three patients required medical therapy (Table 2). The types of atrial tachycardia were focal automatic atrial tachycardia, focal re-entrant atrial tachycardia, and ectopic atrial tachycardia (Table 2 and Figure 1). All three pediatric patients required medical therapy and were discharged on anti-arrhythmic therapy: one patient (focal automatic atrial tachycardia) on beta-blocker (atenolol), and two patients (focal re-entrant atrial tachycardia and ectopic atrial tachycardia) on beta-blocker (atenolol/sotalol) and flecainide. One patient on mono-therapy with atenolol continued therapy for 6 months with no further recurrence. The therapy was discontinued at 6 months at the time of a follow-up visit. The remaining two patients treated with dual therapy continues with beta-blocker (atenolol and sotalol) after flecainide was discontinued after 6 months; routine Holter monitoring shows occasional non-sustained episodes of atrial tachycardia but no sustained episodes on beta-blocker therapy only.

**Table 2:** Pediatric patients with atrial arrhythmias.

	Mechanism	Age (years)	Onset following LTx (days)	Post LTx hospital stay (days)	Drug therapy	Discharged home on therapy	Therapy duration (months)
Patient 1	Focal automatic	15	9	15	Atenolol	Yes	6
Patient 2	Focal Re-entrant	18	10	20	Atenolol Flecainide	Yes	6 (flecainide)
Patient 3	Ectopic atrial tachycardia	13	10	97	Sotalol Flecainide	Yes	6 (flecainide)

Although not included in our statistical analysis, we identified one additional patient who was 27 years old who developed atrial tachycardia following LTx in our institution. This patient developed atrial flutter with intermittent episodes of atrial fibrillation on a post-operative day 10 requiring cardioversion on two occasions for hemodynamic instability with lower blood pressure. His post LTx echocardiogram showed normal biventricular function. He was discharged home on mono-beta blocker therapy with atenolol, which was discontinued at 6 months.

## Discussion

Not surprisingly, the current analysis found the occurrence of atrial arrhythmias following LTx at a pediatric program at similar rates as reported by other pediatric institutions (13–15). Compared to the largest experience of atrial arrhythmias in pediatric LTx recipients recently published, differences for our cohort were older age (older than 12 years old) and the need for intervention in all patients with atrial arrhythmias which all occurred within 10 days following LTx (15).

Focal automatic, re-entrant, and ectopic atrial tachycardia were observed among pediatric patients with all arrhythmias occurring within 10 days following LTx. All three pediatric patients had structurally normal hearts with normal biventricular function with trivial tricuspid regurgitation and no mitral regurgitation on echocardiograms following LTx. Two of the three patients were managed with dual therapy (flecainide and beta-blocker); flecainide was discontinued at 6 months and beta-blocker has been continued with no sustained episodes of atrial arrhythmias. The remaining patient was treated with mono beta-blocker therapy for 6 months without any recurrence.

Although not included in the statistical analysis, there was one additional 27-year-old patient who developed atrial flutter with fibrillation 10 days following LTx who required a cardioversion as well as medical therapy with atenolol. Beta blocker therapy was discontinued after 6 months with no further recurrence. With the occasional need for young adult patients requiring LTx to be performed at our institution, we believe it is important at least to mention all patients as this may need to occur at other pediatric institutions. Although a vast majority of LTx recipients at the pediatric LTx centers are under the age of 18, it is not uncommon for young adults over the age of 18 with childhood-onset conditions to continue their care at the pediatric hospitals as those patients' lifespan increases. About 10% of LTx volume performed at the pediatric LTx centers have been for patients whose ages range from 18 to 34 years old based on the Organ Procurement and Transplantation Network data dashboard (21). As the demand for caring and managing young adults with childhood-onset diseases in the pediatric hospitals increases, it is also increasingly important to take into account their hospital course and outcome at the pediatric centers to improve overall patient care.

Due to surgical and medical advancements as well as improved organ matching for pediatric patients, post-LTx outcomes in children and young adults have slowly improved. Numerous studies have shown that fewer complications during the early post-LTx course is crucial for long-term survival (2–4, 7, 22). Common post-operative complications directly involve the lung allograft including primary graft dysfunction, bleeding, and infection, while extra-pulmonary complications include acute kidney injury, atrial arrhythmias, and stroke (2–5, 7, 22). Among adult LTx recipients, the prevalence of early atrial arrhythmia following LTx is comparable to that following heart transplant, which is up to 50% and is associated with increased mortality and morbidity (8–10, 12). In previous studies among pediatric LTx recipients, supraventricular tachycardia along with atrial flutter and fibrillation, ventricular tachycardia, junctional rhythm, and heart block are reported arrhythmias (14–16). Previous literature reported that most of these arrhythmias were transient and did not require long-term therapy. The patients with atrial flutter requiring medical treatment only received treatment for 6 months post-LTx without recurrence (16). Our current analysis identified similar findings in our cohort. As we consider future management of children and young adults undergoing LTx at our institution, we are being proactive in future management. Since focal re-entrant atrial tachycardia and atrial flutter can be terminated by rapid atrial pacing, placement of temporary atrial wires could potentially be useful in those recipients deemed at higher risk.

The underlying mechanisms for developing atrial arrhythmias following LTx are considered to be multi-factorial including left atrial incisions and associated suture lines of the pulmonary venous anastomosis and myocardial swelling/edema after surgery with increased inflammation and fluid overload (10, 12). Increased sympathetic activity following surgery from post-operative pain and/or inotropes use has been thought to contribute to the incidence of atrial tachycardia as well as electrolytes disturbances following surgery (10, 12). A combination of these common post-surgical changes could all play a role in developing atrial tachycardia especially in the early stage of post-operative period.

All our patients with atrial arrhythmias had normally structured hearts with normal biventricular function indicating arrhythmias were not related to cardiac anatomy or function. Risk factors identified for developing atrial arrhythmias among adult recipients include older age, history of coronary artery disease, smoking, hypertension, and male gender (8–10, 12). The typical occurrence of atrial arrhythmia among adult recipients is within seven days following LTx (8–10). Common interventions for atrial arrhythmia in adult LTx recipients include cardioversion and beta-blocker therapy. Although atrial arrhythmia among adult lung recipients are associated with increased mortality following LTx, there is no clear evidence supporting a similar association in pediatric LTx recipients as noted in the current analysis and recently published data by our colleagues at other pediatric institutions.

One of main limitations of the current analysis was the small sample size. Although our cohort is smaller than a recent study that enrolled 91 pediatric LTx recipients and a few limited other pediatric studies of similar or smaller sample size, our analysis presents very distinct clinical courses and interventions required (15). In that regard, we believe data presented in the current analysis provides a timely and much-needed addition and further insights to the nascent growth of studies investigating atrial arrhythmia in LTx recipients at pediatric centers. Other limitations of our study include a short follow-up period of less than 2 years post-LTx warranting a future study with longer impact of atrial tachycardia following LTx in pediatric patients.

## Conclusion

Atrial arrhythmia following LTx is a less common complication after LTx in children compared to adults. The occurrence of atrial arrhythmia appears to be similar in the LTx population across pediatric institutions, but the current analysis discovered different presentations and interventions needed compared to other pediatric studies. With such small patient populations across all pediatric LTx programs in the United States and variability in presentation and therapeutic interventions in such early post-operative complications, such as atrial arrhythmia, we believe this analysis further supports the need of the respective pediatric LTx programs to work together in an effort to increase sample sizes and extend follow-up periods to determine how to better define post-LTx complications. We believe this collaborative approach would facilitate mitigation of early post-operative complications and improve longer-term outcomes for children and young adults.

## Data Availability

The raw data supporting the conclusions of this article will be made available by the authors, without undue reservation.
